# 565. Nationwide trends in respiratory syncytial virus (RSV) immunizations and RSV immunization efficacy in children aged 2 and under

**DOI:** 10.1093/ofid/ofaf695.174

**Published:** 2026-01-11

**Authors:** Kathryn Lang, David Alfego, Min Kyung Lee, Laura Gillim, Charles M Walworth, Suzanne Dale, Colm Smart, Ruth Carrico, Payman Ghasemi

**Affiliations:** VaxCare, San Marcos, CA; Labcorp, Burlington, North Carolina; Labcorp, Burlington, North Carolina; Labcorp, Burlington, North Carolina; Monogram Biosciences/LabCorp, Laguna Beach, CA; Labcorp, Burlington, North Carolina; VaxCare LLC, Hooksett, New Hampshire; Norton Healthcare, Louisville, Kentucky; VaxCare, San Marcos, CA

## Abstract

**Background:**

Nirsevimab, a monoclonal antibody against respiratory syncytial virus (RSV), has been recommended for infants under 8 months born to women unvaccinated for RSV and for children aged 8 – 19 months with risk of severe RSV infection. Nirsevimab has been shown to offer protection for ∼5 months. We investigate RSV immunization and diagnostic testing in children aged 2 and under since its approval in mid-2023.

**Figure 1:**
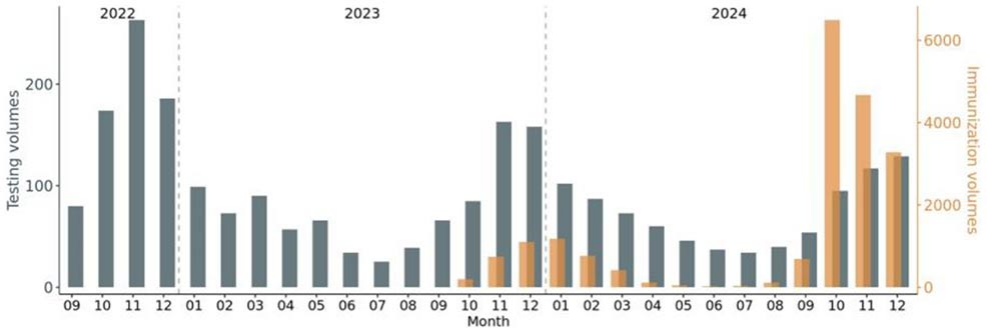
Monthly RSV immunizations and testing volumes in children aged 2 and under from September 2022 to December 2024. Gray bars and left y-axis indicate RSV testing volumes. Orange bars and right y-axis indicate RSV immunization volumes.

**Figure 2:**
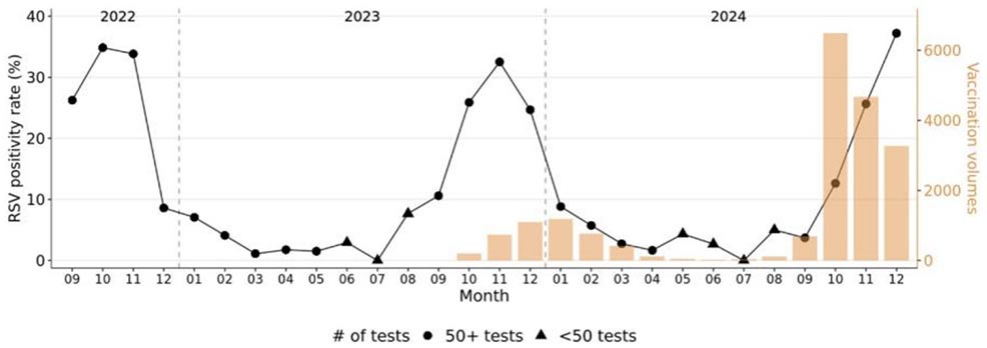
Monthly RSV positivity rates and immunization volumes in children aged 2 and under from September 2022 to December 2024. Line plot indicates RSV positivity rates. Orange bars and right y-axis indicate RSV immunization volumes. Shapes indicate the monthly number of diagnostic tests performed.

**Methods:**

A crossover analysis of two nationwide datasets of 19,842 RSV immunization events from October 2023 to December 2024 and 2,532 RSV diagnostic tests from September 2022 to December 2024 was performed for children aged 2 and under who received seasonal vaccinations. Time-to-event analysis for infants under 8 months with RSV diagnostic test within one year of RSV immunization was conducted.

**Figure 3:**
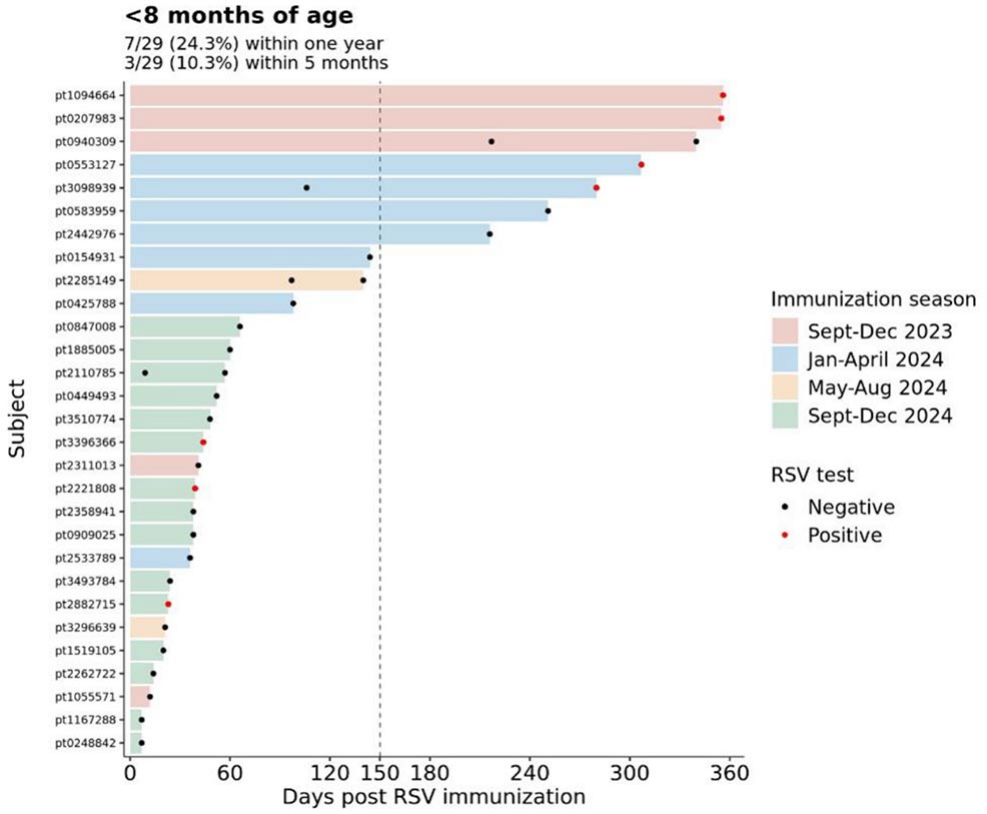
RSV diagnostic testing results within one year of RSV immunization in 29 infants under 8 months of age. Bars are colored by when each patient received the RSV immunization.

**Figure 4:**
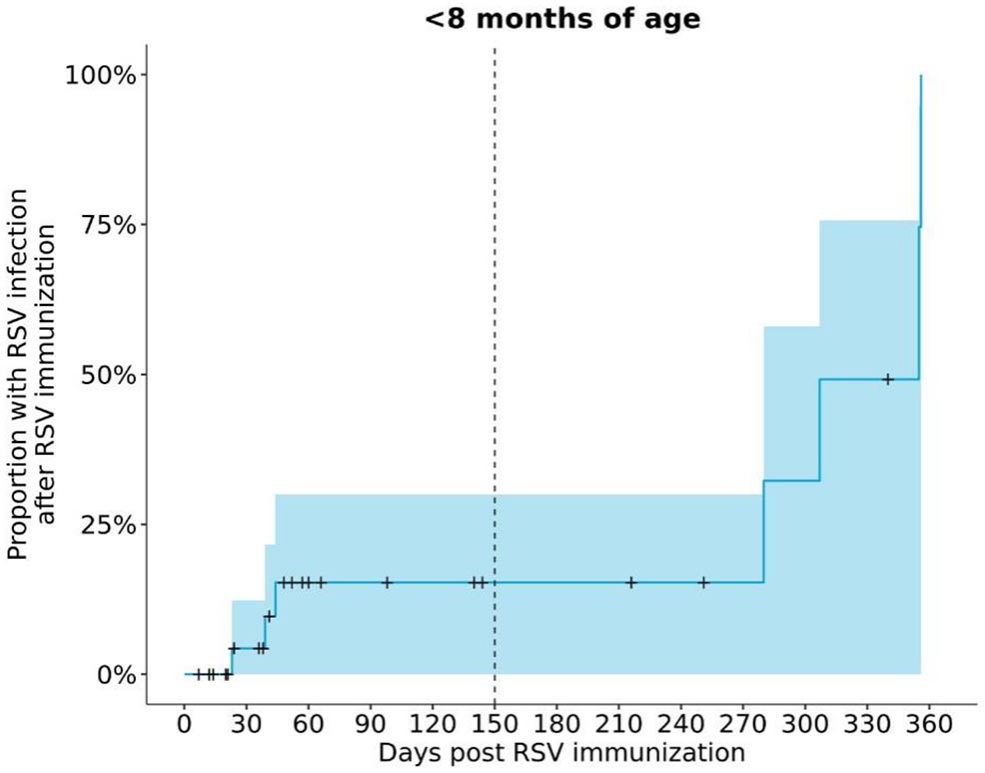
Cumulative incidence of RSV infection after RSV immunization in infants under 8 months of age (N = 29). Patients were censored at their last RSV diagnostic test.

Cumulative incidence of RSV infection after RSV immunization in infants under 8 months of age (N = 29). Patients were censored at their last RSV diagnostic test.

**Results:**

Peak RSV testing occurred in November 2022 and 2023, and in December 2024 in children aged 2 and under (Fig 1). Peak RSV immunizations occurred in January during the 2023 – 2024 RSV immunization season and in October during the 2024 – 2025 RSV immunization season. Peak RSV immunizations occurred two months prior to peak RSV diagnostic testing months in 2023 – 2024 and 2024 – 2025 RSV immunization seasons. Highest RSV positivity rates were observed two months prior to peak RSV immunization month in the first RSV immunization season (Fig 2). In contrast, peak RSV immunization occurred prior to peak RSV positive month in the second RSV immunization season. 7 infants (24.3%) and 3 infants (10.3%) had positive RSV tests within one year and within five months of RSV immunization, respectively, among 29 infants under 8 months of age (Fig 3). The cumulative incidence of breakthrough RSV infection was 15.3% at 150 days after immunization (95% CI = 0.0 – 30.0%, Fig 4).

**Conclusion:**

More children aged 2 and under received the RSV immunization earlier in 2024 versus 2023, reflecting the rolling adoption of the new RSV immunization schedule and recommendations. Both early breakthrough RSV infections and infection greater than 6 months since immunization were observed post RSV immunization in this limited dataset. As such, RSV testing should be considered for children even in the presence of documented RSV immunization.

**Disclosures:**

All Authors: No reported disclosures

